# Retraction Note: Nuciferine inhibits the progression of glioblastoma by suppressing the SOX2-AKT/STAT3-Slug signaling pathway

**DOI:** 10.1186/s13046-023-02662-9

**Published:** 2023-04-20

**Authors:** Zizhuo Li, Yaodong Chen, Tingting An, Pengfei Liu, Jiyuan Zhu, Haichao Yang, Wei Zhang, Tianxiu Dong, Jian Jiang, Yu Zhang, Maitao Jiang, Xiuhua Yang

**Affiliations:** 1 Department of Abdominal Ultrasound, The First Affiliated Hospital of Harbin Medical University, 150001 Harbin, People’s Republic of China; 2 Department of Magnetic Resonance, The First Affiliated Hospital of Harbin Medical University, 150001 Harbin, People’s Republic of China; 3 Department of Pathology, The First Affiliated Hospital of Harbin Medical University, 150001 Harbin, People’s Republic of China

**Retraction Note:** ***J Exp Clin Cancer Res*** **38, 139 (2019)**

**https://doi.org/10.1186/s13046-019-1134-y**

The Editor-in-Chief has retracted this article. Concerns have been raised about Fig. 3a, specifically:

- The 0h control panels for U87 and U251 appear to be identical
- The 0h NF 25µM panels for U87 and NF 25µM appear to be identical
- The 0h NF 30 µM panels for U87 and U251 appear to partially overlap
- The 24h control panels for U87 and U251 appear to be identical
- The 24h NF 25 µM panels for U87 and U251 appear to partially overlap
- The 24h NF 30 µM panels for U87 and U251 appear to partially overlap
- The 48h control panels for U87 and U251 appear to partially overlap
- The 48 NF 25 µM panels for U87 and U251 appear to be identical

The Editor-in-Chief therefore no longer have confidence in the results and conclusions of this article.

Authors Zizhuo Li and Xiuhua Yang agree with this retraction. The other authors have not responded to correspondence regarding this retraction.

